# A medium‐density genotyping platform for cultivated strawberry using DArTag technology

**DOI:** 10.1002/tpg2.20399

**Published:** 2023-11-08

**Authors:** Michael A. Hardigan, Mitchell J. Feldmann, Jason Carling, Anyu Zhu, Andrzej Kilian, Randi A. Famula, Glenn S. Cole, Steven J. Knapp

**Affiliations:** ^1^ USDA‐ARS, Horticultural Crops Production and Genetic Improvement Research Unit Corvallis Oregon USA; ^2^ Department of Plant Sciences University of California Davis Davis California USA; ^3^ Diversity Arrays Technology University of Canberra Bruce Australian Capital Territory Australia

## Abstract

Genomic prediction in breeding populations containing hundreds to thousands of parents and seedlings is prohibitively expensive with current high‐density genetic marker platforms designed for strawberry. We developed mid‐density panels of molecular inversion probes (MIPs) to be deployed with the “DArTag” marker platform to provide a low‐cost, high‐throughput genotyping solution for strawberry genomic prediction. In total, 7742 target single nucleotide polymorphism (SNP) regions were used to generate MIP assays that were tested with a screening panel of 376 octoploid *Fragaria* accessions. We evaluated the performance of DArTag assays based on genotype segregation, amplicon coverage, and their ability to produce subgenome‐specific amplicon alignments to the FaRR1 assembly and subsequent alignment‐based variant calls with strong concordance to DArT's alignment‐free, count‐based genotype reports. We used a combination of marker performance metrics and physical distribution in the FaRR1 assembly to select 3K and 5K production panels for genotyping of large strawberry populations. We show that the 3K and 5K DArTag panels are able to target and amplify homologous alleles within subgenomic sequences with low‐amplification bias between reference and alternate alleles, supporting accurate genotype calling while producing marker genotypes that can be treated as functionally diploid for quantitative genetic analysis. The 3K and 5K target SNPs show high levels of polymorphism in diverse *F*. × *ananassa* germplasm and UC Davis cultivars, with mean pairwise diversity (π) estimates of 0.40 and 0.32 and mean heterozygous genotype frequencies of 0.35 and 0.33, respectively.

## Core Ideas

- Routine deployment of genomic selection (GS) requires access to cost‐effective genotyping tools and technologies.
- We present a medium‐density genotyping platform with 3k and 5k subgenome‐specific sites for octoploid strawberry.
- The core sites cover the FARR1 octoploid reference genome and accurately captures genomic relatedness for GS.

**Abbreviations**
GEBV: genome estimated breeding value
BLUP: best linear unbiased prediction
GBLUP: genomic best linear unbiased prediction
EMM: estimated marginal mean
LMM: linear mixed model
MAS: marker‐assisted selection
GS: genomic selection
MIPs: molecular inversion probes
SNP: single nucleotide polymorphism
HRM: high‐resolution melt
GBS: genotyping‐by‐sequencing
VCF: variant call format
DArT: Diversity Array Technology Ltd.
WGS: whole genome shotgun
OTV: off‐target variant
KASP: Kompetitive Allele Specific PCR
ASV: average semivariance
MAF: minor allele frequency
GRM: genomic relationship matrix
RMSE: root mean square error.

## 1 INTRODUCTION

Public breeding programs have made significant progress toward the development and application of molecular breeding tools for allo‐octoploid (2*n*=8x=56) garden strawberry (*Fragaria* × *ananassa* Duch.) in recent years Whitaker et al. (2020). The publication of multiple single nucleotide polymorphism (SNP) arrays for strawberry from 2015 to 2020 enabled genetic and physical mapping of new loci controlling traits related to disease resistance and fruit quality (Bassil et al., 2015; Barbey et al., 2021; Castillejo et al., 2020; Jiménez et al., 2023; Noh et al., 2018; Nelson et al., 2021; Nellist et al., 2019; Oh et al., 2019; Osorio et al., 2021; Oh et al., 2021; Pincot et al., 2018, 2020, 2021, 2022; Pillet et al., 2017; Petrasch et al., 2022; Salinas et al., 2019). Progress in this area has only increased following the publication of multiple high‐quality, chromosome‐scale assemblies of octoploid Fragaria genomes in the last 5 years (Edger et al., 2019; Hardigan et al., 2021; Lee et al., 2021; Mao et al., 2023; Shirasawa et al., 2021). These genome assemblies, in combination with resequencing, genomic variant calling, and linkage mapping have demonstrated that strawberry contains mainly diploid‐behaving subgenomes with sufficient sequence divergence, in many cases, to allow targeting of specific homeologous sites for marker genotyping and, potentially, future CRISPR editing (Hardigan et al., 2020, 2021; Ledda et al., 2019). With this new landscape for quantitative genetic and functional genomic research in octoploid *Fragaria*, the outlook for molecular breeding applications has improved significantly compared to only a decade ago when the first diploid strawberry genome sequence was published (Shulaev et al., 2011).

Recent mapping studies supported by improved genomes and gene annotations have uncovered large‐effect genetic loci controlling traits of high economic value to the strawberry industry such as resistance to multiple races of soil‐borne pathogen *Fusarium oxysporum* f. sp. *fragariae* (Pincot et al., 2018, 2022). The design of Kompetitive Allele Specific PCR (KASP) or high‐resolution melt (HRM) assays to screen for major resistance genes in large populations is now within reach of breeding programs. These technologies are efficient and cost‐effective for marker‐assisted selection (MAS) when targeting small numbers of genetic loci controlling a large fraction of variance for a given trait (Castillejo et al., 2020; Noh et al., 2018; Oh et al., 2021; Pincot et al., 2022; Verma et al., 2018; Whitaker et al., 2020). However, small marker panels are unable to capture broad allelic diversity across large numbers of haploblocks in the approximately 800‐Mb strawberry genome. Other economically important strawberry traits have been shown to be highly quantitative and controlled by many genetic loci with individually small effects (Gezan et al., 2017; Jiménez et al., 2023; Osorio et al., 2021; Pincot et al., 2020; Petrasch et al., 2022). Genomic prediction has the potential to improve selection efficiency for heritable but genetically complex traits over multiple generations and has part of the molecular breeding toolbox for parent and seedling selection in strawberry (Gezan et al., 2017; Osorio et al., 2021; Whitaker et al., 2020). Pincot et al. (2020) demonstrated the viability of genomic prediction with subgenome‐targeting genetic markers in selecting for quantitative resistance to wilt caused by *Verticillium dahliae* Klebahn, for which no major sources of resistance were identified in 984 wild and cultivated strawberry accessions. As open‐source and increasingly user‐friendly tools for genomic prediction continue to be developed, affordable genotyping platforms are required to support their implementation in breeding pipelines.

**Core Ideas**

- Routine deployment of genomic selection (GS) requires access to cost‐effective genotyping tools and technologies.
- We present a medium‐density genotyping platform with 3k and 5k subgenome‐specific sites for octoploid strawberry.
- The core sites cover the FARR1 octoploid reference genome and accurately captures genomic relatedness for GS.

Current high‐density marker platforms such as SNP arrays are too costly for many public breeding programs to use for genotyping populations containing hundreds to thousands of individuals, posing a barrier to the introduction of genomic selection into the annual breeding cycle. The availability of low‐cost, high‐throughput, and mid‐density genetic marker screening platforms may assist in the adoption of genomic prediction and selection in polyploid specialty crop systems including strawberry (Ariza‐Suarez et al., 2022; Cerioli et al., 2022; Oh et al., 2019; Raman et al., 2022; Semalaiyappan et al., 2023; Wang et al., 2018, 2023). We developed and tested a panel of molecular inversion probe (MIP) marker assays to deploy with the “DArTag” genotyping service offered by Diversity Arrays Technology Ltd. (DArT; Canberra, Australia) in order to provide a low‐cost, mid‐density marker genotyping solution suitable for genomic prediction in strawberry. From the total pool of tested DArTag assays, we identified and selected 3000 and 5000 (3K and 5K) marker panels as DArTag production sets that can be used by strawberry research programs, or selectively mixed and combined with other validated assays to generate new marker panels in the future.

We approached the challenge of developing and selecting assays in strawberry with the goal of balancing genome‐wide representation of allelic diversity with technical performance regarding subgenome‐specific and copy‐specific sequence amplification. MIP amplicon sequencing is an effective strategy for re‐genotyping the same target loci, offering reproducibility across genotyping runs and between studies utilizing the same platform (Almomani et al., 2020; Pérez Millán et al., 2018; Wang et al., 2012). Amplicon‐based genotyping in an allo‐octoploid also poses an increased risk of off‐target sequence amplification and, as a result, the merging of ancestrally related but independently recombining DNA variation from up to four subgenomes into the output of a single marker. Strawberry's four subgenomes appear to recombine independently and, as a result, can be treated as functionally diploid for the purpose of quantitative genetic analysis (Edger et al., 2019; Hardigan et al., 2020, 2021). Therefore, we prioritized the identification of subgenome‐specific and diploid‐behaving probe designs to avoid creating a genotyping platform populated with variable‐dosage markers that may confuse downstream analyses, artificially inflate estimates of allelic diversity at target loci, and conflate estimates of allele frequencies across subgenomes when functional mutations occur on specific subgenomes. We sought to ensure the suitability of targeted assays for two potential genotyping scenarios: (1) basic counting of reference and alternate allele‐matching sequences inferred from the reference design region without alignment information, and (2) variant calling by aligning amplicon sequences to a reference genome and performing alignment‐based variant calling to produce a variant call format (VCF) file, as with genotyping‐by‐sequencing (GBS) or whole‐genome resequencing approaches. The second approach requires additional bioinformatic steps but offers the benefit of parsing sequences derived from off‐target amplification in polyploid backgrounds to their respective subgenomic loci, supporting genotyping of additional background SNPs and indels flanking the target SNP sites, that is, novel allele discovery.

We demonstrate that the selected 3K and 5K DArTag panels generate amplicons that support unique, subgenome‐specific alignments to the FaRR1 genome assembly and subsequent disomic genotype calls with low amplification bias between the reference and alternate alleles. The target SNPs provide physical coverage of the A, B, C, and D subgenomes with low subgenome bias in the 3K panel and slightly higher bias in favor of the A subgenome in the 5K panel due to the availability of more QC‐passing assays. The 3K and 5K target SNPs show high levels of polymorphism in heirloom and commercial *F*. × *ananassa* accessions. Wild diversity estimates were approximately 25% lower at target SNPs due to our prioritization of domesticated strawberry polymorphism for genomic prediction; however, we confirmed that the DArTag panels are still able to resolve significant genome‐wide polymorphism in wild accessions. An additional 9768 validated SNP and indel sites were identified in amplicon sequences flanking the target SNPs. These may provide incrementally higher resolution of genotypic diversity, although we observed that nucleotide diversity at these sites was generally lower than target SNPs. The 3K and 5K DArTag panels will be tested in the future for genomic prediction accuracy in strawberry populations containing hundreds to thousands of individuals. While offering lower genotyping resolution compared to existing SNP arrays (Bassil et al., 2015; Hardigan et al., 2020; Verma et al., 2017), the mid‐density DArTag panels should allow for genomic prediction at a fraction of the cost while providing a similarly effective tool for applications such as fingerprinting and pedigree validation.

## 2 MATERIALS AND METHODS

### 2.1 Plant material and DNA extraction

The diversity panel of 145 octoploid *Fragaria* accessions used to perform whole genome shotgun (WGS) short read alignments to FaRR1 and variant calling to identify SNP candidates for probe design were sequenced and described by Hardigan et al. (2021). We assembled a screening panel of 376 octoploid *Fragaria* accessions to use for testing the performance of the DArTag probes constructed by DArT for the submitted SNP candidates (Table S1). The screening panel consisted of three bi‐parental full‐sib mapping populations with 92–95 progeny, and the remaining 96 accessions comprised a mixture of UC Davis cultivars (*n* = 22) and advanced selections (*n* = 27), diverse historic UC Davis and global *F*. × *ananassa* cultivars (*n* = 34), and wild accessions (*n* = 13). Immature leaf tissue was collected from greenhouse and field‐grown plants for genomic DNA extraction. Leaf samples were placed into 1.5‐mL tubes or envelopes and lyophilized in a Benchtop Pro (VirTis SP Scientific, Stone Bridge, NY). Approximately 200 mg of lyophilized leaf tissue from each sample was placed in 2.0‐mL 96‐well deep‐well plates with stainless steel beads and ground in a Mini 1600 (SPEX Sample Prep, Metuchen, NJ). Genomic DNA was extracted from the ground leaf samples using the E‐Z 96^®^ Plant DNA Kit (Omega Bio‐Tek, Norcross, GA, USA). The protocol was modified by adding Proteinase K to the lysis buffer for a 0.2 mg/mL concentration and increasing the lysis incubation step to 45 min at 65°C. Once the lysate separated from the cellular debris, RNA was removed by adding RNase A. The mixture was incubated at room temperature for 5 min before a final spin down. The sample was incubated at 65°C for 5 min after adding the elution buffer. DNA quantification was performed using Quantiflor dye (Promega, Madison, WI) on a Synergy HTX (Biotek, Winooski, VT).

### 2.2 Genomic variant calling of candidate marker sites

We obtained Illumina short‐read sequences for the panel of 145 octoploid *Fragaria* accessions described in Hardigan et al. (2021). Sequences were downloaded from the NCBI Sequence Read Archive under BioProject PRJNA578384 using the “fastq‐dump” command from the SRA‐Toolkit software (3.0.2). Sequence reads were aligned to the FaRR1 strawberry genome (Hardigan et al., 2021) using BWA‐MEM (v7.17) with default parameters and piped into BAM format alignment files using SAMTools (v1.16.1) (Danecek et al., 2021; Li, 2013). We used PicardTools (2.7.1) to mark PCR and optical duplicates and used GATK (3.8) to perform indel realignment in the BAM files (McKenna et al., 2010). We used the FaRR1 alignments for 145 octoploid *Fragaria* accessions to predict SNP and indel sites in the FaRR1 assembly with the haplotype‐caller software FreeBayes (v9.9.2) (Garrison & Marth, 2012). Only read alignments with MapQ values above 30 and percent alignment identity above 90% and aligned bases with Phred quality above 20 were used by FreeBayes. We used vcflib (v1.0.3) to impose a set of hard filters on the VCF file produced by FreeBayes (Garrison et al., 2022). This included removing variants with minor allele frequencies below 0.025 (vcflib: AF ≥ 0.025& RPR ⩽ 0.975), low site quality (vcflib: QUAL > 1000), low contribution of alleles to site quality (vcflib: QUAL/AO > 10), low read coverage (vcflib: DP > 500), strand bias (vcflib: SAF > 0& SAR > 0), read‐placement bias (vcflib: RPP ≥ 3& RPR ≥ 3), unbalanced mapping quality of reference and alternate alleles (vcflib: 0.4 ⩽ [MQM/MQMR] ⩽ 2.5), low end‐placement probability score (vcflib: EPP ≥ 3), and low strand‐bias probability score (vcflib: SRP ≥ 3 & SAP ≥ 3). We also excluded alleles that showed highly biased allele balance at heterozygous sites (vcflib: AB ≥ 0.2 & AB ⩽ 0.8).

### 2.3 Evaluation of candidate marker sites

We extracted 200 bp design sequences flanking FaRR1 candidate variant sites using BEDTools (v2.30.0) “getfasta” command (Quinlan & Hall, 2010). Target sequence MIP probe scoring was performed by running MIPgen (v1.1) with the “‐snp_file” command designating a VCF file of off‐target variant (OTV) sites (Boyle et al., 2014). Target sequence uniqueness metrics were based on alignments of design sequences to FaRR1 generated using BLAST (v2.9.0+), running “blastn” with default parameters, and retaining alignments with minimum 60% query coverage (Camacho et al., 2009). OTV interference probability metrics were estimated by counting the allele frequencies of flanking OTVs in the design sequence identified using BEDTools “intersect” command. Sequence repetitiveness metrics were estimated by running RepeatMasker (v4.1.4) on the FaRR1 assembly with default parameters (Chen, 2004). Marker polymorphism based on target site pairwise nucleotide diversity was estimated the using VCFtools “–site‐pi” command (v0.1.16) (Danecek et al., 2011).

### 2.4 DArTag marker genotyping

MIP probe marker genotypes for target SNPs were estimated using an amplicon counting approach with amplicon count data generated in the DArT reports (Files S2 and S3). The amplicons which align to the target sequence are assessed for membership to the target locus using the full sample set (as a single locus with disomic inheritance), and those which are determined to belong are used for calling. The frequency of amplicons matching the reference or alternate allele at the target SNP was evaluated for each sample. Samples for which ≥95% of amplicons matched the reference or alternate allele were scored as “AA” and “BB” homozygous genotypes, respectively, while samples for which ≥20% of amplicons matched the reference allele and ≥20% of amplicons matched the alternate allele were scored as “AB” heterozygous genotypes. Samples with fewer than 10 amplicons or ambiguous amplicon dosages were scored as missing genotypes. Marker genotypes for target SNPs and OTVs were re‐estimated by performing genomic variant calling with the DArTag amplicon sequence alignments to the FaRR1 assembly. The raw MIP probe amplicon sequences were obtained from DArT, which included a file in FASTQ format for each of the 376 accessions in the screening panel. We used CutAdapt (v4.1) to clip the 5‐prime sample barcode (‘‐g CGATGGATCCAGTGC') and any remaining DArTag adapter sequences (‘‐b ACCGATCTCGTATGCCGTCTTCTGCTTGAAAAAAAAAA ‐b TTTTTTTTTTCAAGCAGAAGACGGCATACGAGATCGGT') from the raw amplicon reads, while filtering low‐quality bases, short sequences, and sequences containing too many ambiguous base calls (‘–times 4 ‐O 5 ‐e 0.125 ‐q 15 ‐m 30 –max‐n 5'). The barcode and adaptor‐trimmed amplicon sequences were aligned to FaRR1 (Hardigan et al., 2021) using BWA‐MEM (v7.17) (Li, 2013) with default parameters, and alignments were converted to BAM format using SAMTools (v1.16.1) (Danecek et al., 2021). We used alignments to FaRR1 for the 376 screening panel accessions to genotype target SNPs and OTVs within the FaRR1 MIP design regions using FreeBayes (v9.9.2) (Garrison et al., 2022). Only read alignments with MapQ values above 30 and percent alignment identity above 90% and aligned bases with Phred quality above 20 were used by FreeBayes. In order to support amplicon‐based genotyping, FreeBayes was run using the “–use‐duplicate‐reads” command and the “–variant‐input” command with a list of variant sites and alleles identified in the WGS diversity panel and the “–only‐use‐input‐alleles–report‐monomorphic” options to enforce reporting of target SNPs and OTVs corresponding to pre‐validated variant sites within the design regions.

### 2.5 Estimating linkage groups and genetic maps

Marker genotypes for the three bi‐parental mapping populations in the 376‐accession screening panel (16C104, 17A975, 18A892) and their respective parent accessions were extracted from the screening panel VCF file and converted to Onemap format. Marker genotype data were analyzed in R using the software package “onemap” (Taniguti et al., 2022). Markers were initially used to calculate 2‐point recombination frequencies using the “ref_2pts()” function and grouped into linkage groups using the “group()” function with a LOD threshold of 6 and a maximum recombination fraction of 0.25. Parent‐specific heterozygous marker genotypes (AA × AB, AB × AA) were used to assign linkage groups and their associated markers to the respective maternal or paternal accession. Marker genotypes segregating in both parental backgrounds (AB × AB) were ignored. Marker orders within each linkage group were determined using the rapid chain delineation algorithm as implemented in the “rcd()” function. The resulting marker orders were used to calculate the genetic maps using the “map()” function.

### 2.6 Estimating genomic relatedness and principal components

Marker genotypes for 279 accessions that had genotypic data from the FanaSNP 50K array (Hardigan et al., 2020) and the strawberry DArTag 3K and 5K DArTag panels in the present study were used to calculate the average semivariance genomic relationship matrix (Feldmann et al., 2022). We used rrBLUP::A.mat() to filter all marker sets’ SNPs for a minor allele frequency (MAF) > 0.05 (Endelman, 2011). In total, we used five different marker sets: (1) 44,891 markers from the FanaSNP 50K SNP array, (2) 6017 target and OTV SNPs from the DArTag 3K panel, (3) 2935 target SNPs from the DArTag 3K panel, (4) 9806 target and OTV SNPs from the DArTag 5K panel, and (5) 4884 target SNPs from the DArTag 5k platform. Subsequently, we used stats::prcomp() to calculate the first principal component of each of the five genomic relationships. All visualizations were created using ggplot2 v3.4.2 (Villanueva & Chen, 2019) and patchwork v1.1.2 (Pedersen, 2022).

## 3 RESULTS AND DISCUSSION

### 3.1 Target variant selection and probe design

We used the WGS panel described by Hardigan et al. (2021) and containing sequences from 145 wild and domesticated octoploid *Fragaria* accessions to perform read alignments and predict genome‐wide SNPs and indels with the improved FaRR1 genome assembly (Hardigan et al., 2021). We initially predicted 68,073,012 unfiltered variants in the FaRR1 assembly. We identified the pool of potential target variants for marker design by applying VCF hard filtering thresholds to the raw variant calls (see Section 2), in addition to exclusively retaining biallelic SNPs and indels with an MAF greater than 0.05 in domesticated accessions. Indels larger than 3 bp were ignored due to higher potential for genotyping errors and allelic complexity. The resulting set of filtered candidate variants contained 6,425,147 SNPs and small indels.

We generated several metrics for the 6,425,147 candidate variant sites to use as selection criteria reflecting MIP probe availability, the likelihood of subgenome‐specific and copy‐specific probe binding, likelihood of off‐target flanking variants interfering with probe binding, and genotypic diversity in the domesticated strawberry germplasm (Table 1). The 200‐bp genomic sequences flanking each candidate variant site were treated as the potential MIP probe design region for calculating most metrics. We evaluated the ability to predict MIP probes in the design regions to select regions more likely to produce working DArTag probes around the corresponding target variants. We targeted regions containing candidate MIP probes generated by MIPgen with logistic scores above 0.7 that flanked the target SNP (Boyle et al., 2014). MIPgen aligns probe designs back to the reference genome and attempts to incorporate the likelihood of single‐copy probe specificity into probe design scores, potentially increasing the likelihood of selecting subgenome‐specific probe designs (Boyle et al., 2014). The sequence uniqueness of candidate variant design regions was also evaluated to select regions with a greater likelihood of subgenome‐specific and copy‐specific probe binding. We estimated sequence uniqueness by aligning the MIP design regions back to FaRR1 and calculating the number of off‐target regions in the assembly with sequence identity above 80%, the number of off‐target regions with sequence identity above 90%, and the percent sequence identity of the best off‐target alignment.

**TABLE 1:** Statistics used to rank the probe design regions of candidate single nucleotide polymorphism (SNP) sites in cultivated strawberry. All designs were tested against the “UCD Royal Royce” (FaRR1) reference genome.

Marker feature	Metrics evaluated	Description
MIP availability	MIPgen status	Predicted MIPgen assay with minimum Log score of 0.7
	MIPgen best score	Highest Log score of predicted MIPgen assays
Target uniqueness (subgenome specificity)	Off‐target best hit	Percent identity of best off‐target alignment
	Off‐target count (90%)	Number of off‐target alignments in FaRR1 with
		percent identity above 90%
	Off‐target count (80%)	Number of off‐target alignments in FaRR1 with
		percent identity above 80%
OTV interference	OTV frequency sum	Sum of domesticated accession allele frequencies
		for OTVs in design region
	OTV count	Total OTV variants in design region
	OTV indel count	Total OTV indels in design region
Sequence repetitiveness	Repetitive DNA content	Percent of design sequence masked by
		RepeatMasker
Marker polymorphism	Target site diversity	Pairwise diversity of biallelic target site in
		domesticated accessions

Abbreviations: MIP, molecular inversion probe; OTV, off‐target variant.

The degree of OTV sequence polymorphism was evaluated in the candidate variant design regions to select regions with a lower likelihood of probe binding interference caused by polymorphic sites flanking the target variant. The goal of avoiding excessive OTV polymorphism in the MIP design regions was to ensure that MIP probes functioned across diverse germplasm not included in the screening panel and to prevent amplification bias between the reference and alternate alleles resulting from OTV interference in non‐reference haplotypes. We estimated OTV polymorphism based on the total number of OTVs, total OTV indels, and the sum of allele frequencies of all adjacent OTVs identified in the design region. Selecting design regions with low OTV polymorphism correspondingly selects for marker loci with reduced local haplotype diversity, which may reduce the likelihood of recovering multi‐allelic genotype information with methods treating full amplicon sequences as haplotype alleles (Voorrips & Tumino, 2022). Due to potentially high rates of attrition when designing marker assays for allo‐octoploids, we prioritized mitigation of amplification bias between samples or alleles within samples by selecting designs with limited polymorphism flanking the target variant site. The local repetitive DNA content in the candidate variant design regions was assessed using the fraction masked by repeat masking software RepeatMasker (Chen, 2004) as an additional strategy to support probe‐binding specificity. Last, we estimated pairwise nucleotide diversity at the target variant site for domesticated strawberry accessions in order to prioritize marker informativeness in breeding populations.

Candidate variant sites were submitted to DArT for marker conversion in two rounds, with the first round including 2000 target variants and the second round including 7000 target variants (File S1). We employed a heuristic scoring approach to assign MIP target scores to the 6,425,147 candidate variant sites based on the average percentile value of each evaluated metric, giving equal weight to metrics reflecting probe availability, target uniqueness, OTV interference, sequence repetitiveness, and marker polymorphism (Table 1). Candidate variants without a MIPgen prediction with log score of 0.7 or higher were excluded without considering other metrics. If the percentile value for any individual metric was below 80%, we set the MIP target score to the minimum observed percentile value. For the first submission, we divided the physical genome space of the FaRR1 assembly into 2000 non‐overlapping windows of equal size. We selected the best candidate based on the design target score and then by the highest pairwise diversity in the domesticated germplasm for equally high‐scoring candidates. In the second submission, we used an iterative selection approach with progressively shrinking thresholds of minimum window size between existing markers to ensure large physical gaps were filled, even with lower‐scoring candidate variants. For the first iteration, the minimum window size for introducing a new marker was set to the maximum distance between any two markers from the first set of 2000. With each pass through the genome, the minimum window size threshold for introducing a new marker was reduced by 1 kilobase (kb), inter‐marker windows were recalculated based on the updated marker set at the start of a round, and the best available marker was added to windows larger than the size threshold. The process was repeated until 7000 additional candidate variants were selected for marker conversion. In both rounds, the target variants were submitted with corresponding MIP design regions extracted from the FaRR1 assembly and the best‐scoring MIPgen probe design flanking the target variant (File S1). With this approach, we attempted to balance genome‐wide marker distribution, maximum representation of *F*. × *ananassa* allelic diversity at target sites, and increased likelihood of DArTag buildability and subgenome‐specific amplification around target sites. 7742 out of 9000 submitted target variants were converted to DArTag assays and evaluated for performance.

### 3.2 Selection of 3K and 5K DArTag panels

We assembled a 376‐sample screening panel of octoploid *Fragaria* accessions to evaluate the performance of the DArTag assays generated for the target SNPs. The screening panel contained three full‐sib families with at least 90 progeny and the remainder comprised a diversity panel of wild and cultivated strawberry accessions (Table S1). The resulting DArTag reports contained genotype information in the form of the reference allele matching and alternate allele matching amplicon counts for the 376 strawberry accessions (Files S2 and S3).

The variant sites of interest with flanking sequence (File S1) were used as input for DArTag design conversion using the DArTag design pipeline of Diversity Arrays Technology Pty. Ltd. DArTag probe sites flanking the variant positions were chosen considering amplicon construct‐based positioning, sequence composition, and alignment uniqueness in the FaRR1 assembly. Probe site locations were bounded to ensure visibility of the target variant site within the intended amplicon sequencing length of either 54bp or 81b. Potential DArTag probe sites inside the bounded region were selected to provide probe sequence length and composition suitable for DArTag assay chemistry, avoiding repetitive sequence and short poly‐G regions unsuited to oligo synthesis. Based on alignment to FaRR1 reference genome assembly, potential DArTag probe designs were eliminated when multiple aligned loci showed 100% identical sequences within the extension region between the two probes. Through assessment of the number of alignments for candidate probe sites, probe designs with lower predicted cross‐hybridization and off‐target amplification were favored.

The performance of the 7742 reported assays was evaluated using a combination of amplicon count statistics, amplicon count‐based genotype calls, and genotype calls produced by aligning amplicon short‐read sequences to FaRR1. We used amplicon count information in the DArTag reports to calculate the fold‐coverage produced by individual DArTag assays relative to the expected mean coverage across all assays assuming uniform amplification, the median sample amplicon coverage (Figure S1A), and the frequency of low‐coverage samples (fewer than 10 amplicon reads) (Figure S1B). The average median per‐sample amplicon coverage across all tested markers was 105X. The mean rate of low‐coverage samples across all tested markers was 11.7%. We produced parallel sets of genotype calls for a comparison of genotypes based on allelic amplicon counts (DArTag report) and based on alignment of the amplicon short reads to the FaRR1 assembly and performing genomic variant calling. Aligning amplicons to a subgenome‐resolved strawberry genome assembly should assist in parsing mixed homeologous amplicons into their respective loci when off‐target amplification occurs. Moreover, we reasoned that assays with strong concordance between counts‐based genotypes and unique subgenome alignment‐based genotypes would have a lower risk of off‐target amplification than assays with weak concordance. We excluded 1042 of 7742 reported assays from consideration because they failed to reproduce a corresponding FaRR1 genomic variant call with identical alleles at the target SNP site after performing alignment‐based variant calling. The percent concordance between the DArTag report counts‐based genotypes and FaRR1 alignment‐based genotype calls was calculated at target SNP sites for the 376 samples in the screening panel (Figure S1C). Of the 6700 reported assays that reproduced alignment‐based variant calls with identical alleles at the target SNP site, 5753 (85.8%) showed at least 90% concordance between the genotyping approaches and average genotyping concordance was 94%. Alignment‐based variant genotypes for the three full‐sib families were also binned into linkage groups to test whether variant sites associated with the assays were mappable as an indicator of diploid‐behaving genotype segregation—we observed that 4552 total assays contained 8208 variants binned into linkage groups.

**FIGURE 1:**
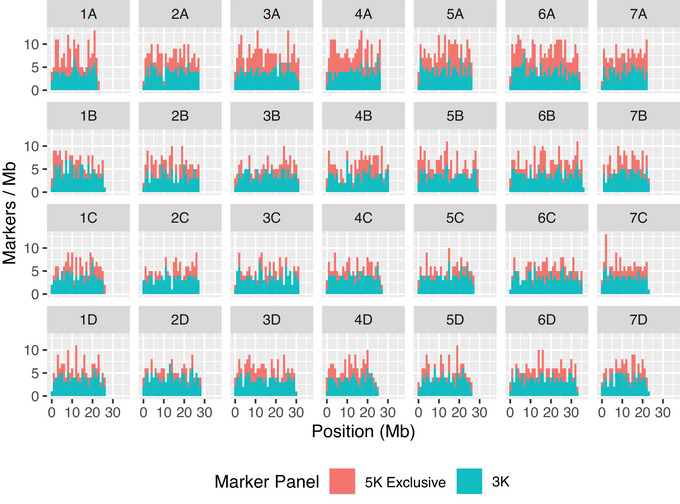
Distribution of marker assays selected for the 3K and 5K DArTag panels in the FaRR1 genome assembly (number of markers per megabase). The 5K‐exclusive markers are those 2000 markers only found in the 5K panel, and the 3K marker panel captures the remaining 3000 markers. The genome is equally well covered by the 3K as the 5K and does not introduce any large gaps on the “UCD Royal Royce” (FaRR1) reference genome.

We selected a set of 5000 filtered assays (5K DArTag panel) by imposing hard filtering thresholds that required a minimum per‐assay amplicon coverage of 10% of the expected mean amplicon coverage, a maximum of 20% samples with low amplicon coverage (<10), a corresponding alignment‐based variant call predicted at the target site with at least 90% genotype concordance, and polymorphic genotype segregation where no single genotype class (AA/AB/BB) accounted for more than 90% of total genotype calls (Table S2). We selected a subset of 3000 assays (3K DArTag panel) from the 5K panel by prioritizing assays with high concordance between the count‐based genotypes and alignment‐based genomic variant genotypes, uniform genomic distribution, and evidence of disomic marker genotype segregation based on linkage group clustering where possible (Table S2). On average, the median per‐sample amplicon coverage for markers selected in the 3K and 5K panels was 118X and 123X, respectively. The mean concordance between count‐based and alignment‐based genomic variant genotypes was 98% in both panels. The distribution of selected markers in the 3K and 5K panels provided genome‐wide physical representation of the four subgenomes (A, B, C, and D) without major gaps in coverage (Figure 1). When assessing total marker counts for the 3K and 5K panels in 1‐Mb non‐overlapping genomic windows, every window contained at least one marker (Figure S2A). The number of markers per window was more uniform for the 3K panel due to using physical distribution information for selection. There were only three instances where the distance between a pair of adjacent markers exceeded 1 Mb for either the 5K or 3K panels, and 96.4% of adjacent marker distances were less than 500 kb in the 3K panel (Figure S2B). The 5K panel showed a greater bias in marker density on the dominant A subgenome compared to the 3K panel, with 36% higher marker density in the A subgenome compared to the B, C, and D subgenomes in the 5K panel, compared to only 8% higher density for the 3K panel. This reflected the lower number of available high‐scoring MIP assays on the non‐dominant subgenomes.

### 3.3 Evaluating marker diversity and sample ascertainment bias

**FIGURE 2:**
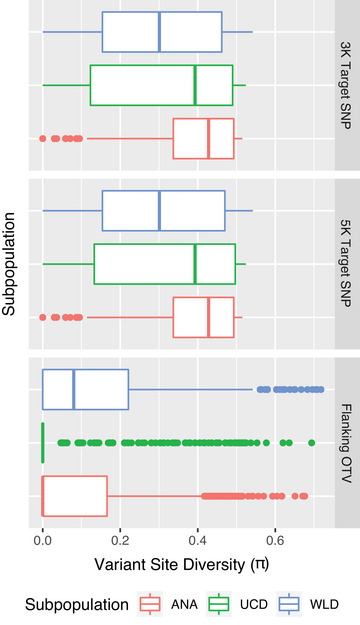
Pairwise nucleotide diversity (π) of 3K panel target single nucleotide polymorphisms (SNPs), 5K panel target SNPs, and flanking off‐target variants (OTVs) in the subpopulations of diverse heirloom *F*. × *ananassa* cultivars (ANA), UC Davis *F*. × *ananassa* cultivars (UCD), and wild octoploid *Fragaria* accessions (WLD). Target SNPs are those SNPs that were specifically targeted from the whole genome shotgun (WGS) data aligned to the “UCD Royal Royce” (FaRR1) reference genome, and flanking OTVs are variants that are discovered within the sequenced amplicon regions.

We prioritized the nucleotide diversity of domesticated strawberry for target variant selection because the intended application of the DArTag panels was genomic prediction in breeding populations. We assessed pairwise diversity (π) in the 3K and 5K panel target SNPs and their flanking OTVs in the subpopulations of the screening panel comprising diverse *F*. × *ananassa* cultivars (*n*=34), UC Davis cultivars (*n*=22), and wild accessions (*n*=13) (Figure 2). The mean pairwise diversity of 5K panel target SNPs was 0.40 for the diverse *F*. × *ananassa* cultivars, 0.32 for UC Davis cultivars, and 0.30 for wild accessions. The mean frequency of heterozygous genotype calls for 5K panel target SNPs was 0.35 for the diverse *F*. × *ananassa* cultivars, 0.33 for the UC Davis cultivars, and 0.15 for wild accessions. Wild accessions are individually expected to be less heterozygous than hybrid cultivars but should contain similar or greater pairwise diversity than diverse *F*. × *ananassa* cultivars (Hardigan et al., 2021). Based on pairwise diversity estimates of 0.30 for wild accessions and 0.40 for diverse *F*. × *ananassa* cultivars, the target variant sites may underestimate wild genetic diversity by at least 25%. The observed level of ascertainment bias may also be inflated due to the small subpopulation size of wild accessions in the screening panel. While the DArTag panel was not designed for comparisons of genome‐wide diversity between domesticated and wild accessions, the target SNPs still capture a sufficient number of wild polymorphic sites to be suitable for evaluating relationships and relative diversity within populations consisting exclusively of wild material.

We observed that a potential benefit of the alignment‐based genotyping approach was the ability to capture additional genotype information from OTVs flanking target variant sites. The 5K panel amplicon regions contained 9768 flanking SNPs and indels with evidence from the WGS panel in addition to the 5000 target SNP sites. By selecting against design regions with high OTV rates in domesticated strawberry, these 9768 flanking variants contained lower pairwise diversity, on average, than the target variant sites (Figure 2). However, many were still polymorphic in the wild accessions and in a subset of the domesticated accessions. Including flanking OTVs when genotyping may mitigate the effect of wild ascertainment bias and provide additional resolution when estimating kinship matrices for genomic prediction. We produced variant site databases (VCF format) containing target SNPs and WGS‐validated OTVs in the 3K and 5K panels for re‐genotyping these sites using an alignment‐based genotyping approach (Files S4 and S5).

We were able to produce low‐density genetic maps using genotypes from the three full‐sib populations included in the screening panel (Table 2; Table S3). The parents included three *F*. × *ananassa* accessions, two *F. virginiana* accessions, and one *F. chiloensis* accession. We produced parent‐specific maps using markers that were heterozygous in a single parent (AB × AA; AA × AB) and retained linkage groups containing at least three markers spanning a minimum of 100 kb in the FaRR1 physical genome. The wild accessions produced genetic maps containing higher genome coverage in fewer linkage groups than the cultivated accessions, despite containing fewer heterozygous marker genotypes. Previous work has shown this is influenced by large runs of homozygosity within individual *F*. × *ananassa* genomes resulting from selection pressure and identity‐by‐descent (Hardigan et al., 2021). These maps demonstrate the ability of selected assays to effectively resolve genome‐wide polymorphism in the octoploid germplasm, including wild species, with low missing genotype data.

**TABLE 2:** Summary of parental genetic maps produced using 5K DArTag panel genotypes for three full‐sib families include the number of linkage groups, the total number of markers in each map, the map size in basepairs estimated using the “UCD Royal Royce” (FaRR1) reference genome, map size in centiMorgans (cM), and percent of the FaRR1 reference genome covered by each map..

Genotype	Species	Linkage groups	Total markers	Map size (bp)	Map size (cM)	Genome coverage (%)
PI 552277	*Fragaria virginiana*	29	1621	721,824,337	2059.4	92.1
PI 612493	*F. virginiana*	28	1627	718,385,397	1961.0	91.6
PI 551753	*F. chiloensis*	29	1253	682,598,434	1463.7	87.1
“Beaver Belle”	*F*. × *ananassa*	30	2242	608,745,316	1456.6	77.6
“Camarosa”	*F*. × *ananassa*	35	2402	480,333,264	1286.5	61.3
12C089P002	*F*. × *ananassa*	38	1404	432,364,767	1067.0	55.1

### 3.4 Evaluating allelic ascertainment bias

**FIGURE 3:**
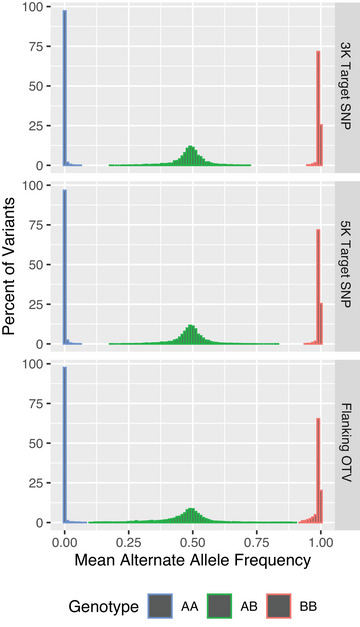
Distribution of mean alternate allele frequencies for homozygous reference (AA), heterozygous (AB), and homozygous alternate (BB) genotype calls for 3K panel target single nucleotide polymorphisms (SNPs), 5K panel target SNPs, and flanking off‐target variants (OTVs) called against the “UCD Royal Royce” (FaRR1) reference genome for cultivated strawberry.

While biological ascertainment bias between wild and cultivated subpopulations or different cultivated subpopulations (i.e., breeding programs) can lead to misinterpretation of total genome‐wide diversity (Heslot et al., 2013; Malomane et al., 2018), technical ascertainment bias in the amplification of homologous allelic sequences increases the likelihood of genotyping errors at loci targeted by markers (Lighten et al., 2014; Zhang et al., 2015). This can result from non‐copy specific oligo binding to both target and off‐target genome sequences, or alternatively, weaker oligo binding to non‐reference haplotypes within on‐target regions due to interference from OTVs around the target SNP. These factors may lead to genotyping errors, ambiguous allele dosages at heterozygous sites, and misclassification of homozygous subgenomic variants as heterozygous when homeologs are co‐amplified. Diploid‐behaving marker assays that target a single subgenome with low amplification bias should produce roughly 1:1 amplicon coverage of the reference and alternate alleles at heterozygous sites. We assessed the mean frequency of reference and alternate allele coverage for homozygous reference (AA), homozygous alternate (BB), and heterozygous (AB) genotype calls across all biallelic SNP genotypes predicted using amplicon alignments to FaRR1 (Figure 3). For target SNPs in the 3K and 5K panels, mean alternate allele frequencies for heterozygous genotypes were in the 40%–60% range for 92.2% and 91.7% of assays, respectively. This predominance of roughly 1:1 reference‐versus‐alternate frequencies at heterozygous sites suggests the selected MIP assays are mainly targeting specific subgenomes with low amplification bias between the reference and alternate allele. The proportion of SNPs with mean alternate allele frequencies in the 40%–60% range fell to 79.3% in OTVs flanking the target sites. Reference and alternate allele coverage may be subject to greater amplification bias at OTVs located farther from the target site, which may pose a greater risk for genotyping error when reporting non‐target SNPs. Moreover, while mean allele frequencies across the 376‐accession screening panel supported low population–wide amplification bias for target SNPs, we observed that a minority of samples in the panel could still exhibit amplification bias on a per‐marker basis. This might occur when undetected, low‐frequency OTVs (rare alleles) occur in the primer binding site. Recently developed software tools such as PolyRAD can correct for overdispersion and amplify bias for genotype calls derived from allelic read counts (Clark et al., 2019). These tools may increase the utility of target and non‐target SNP genotypes by reducing genotyping errors at heterozygous sites with ambiguous allele dosages.

### 3.5 Accuracy of relationships estimated using 3k and 5k DArTag panels

**FIGURE 4:**
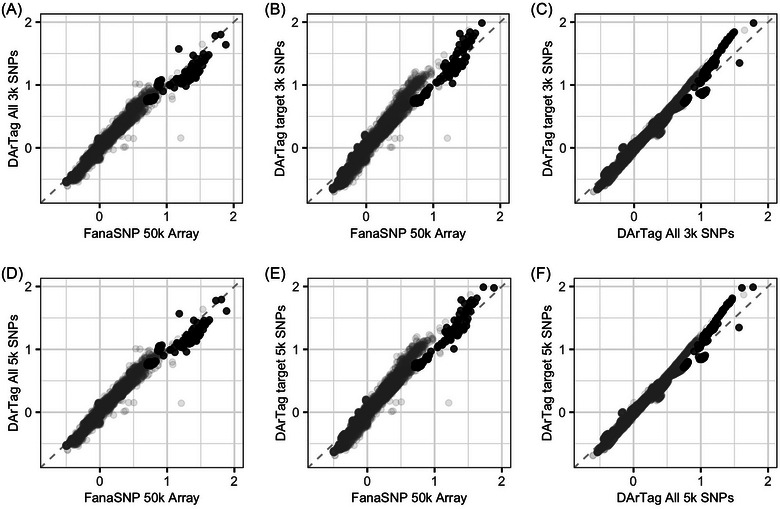
Distribution of genomic relatedness estimated using different marker panels for *n* = 279 diverse cultivated strawberry accessions. (A) Distribution of relatedness estimates using 49,483 FanaSNP markers and 6017 target + off‐target variant (OTV) (All) DArTag 3K panel variants. (B) Distribution of relatedness estimates using 49,483 FanaSNP markers and 2935 target DArTag 3K panel variants. (C) Distribution of relatedness estimates using 6017 target + OTV (All) DArTag 3K panel variants and 2935 target DArTag 3K panel variants. (D) Distribution of relatedness estimates using 49,483 FanaSNP markers and 9806 target + OTV (All) DArTag 5K panel variants. (E) Distribution of relatedness estimates using 49,483 FanaSNP markers and 4884 target DArTag 5K panel variants. (F) Distribution of relatedness estimates using 9806 target + OTV (All) DArTag 5K panel variants and 4884 target DArTag 5K panel variants. In all plots, the gray points are off‐diagonal elements of the genomic relationship matrices (GRMs), and the black points are the diagonal elements of the GRM. SNP, single nucleotide polymorphism.

We found a high degree of similarity between the genomic relationship matrices (GRMs) estimated using five marker sets: (1) 44,891 markers from the FanaSNP 50K SNP array, (2) 6017 target and OTV SNPs from the 3K DArTag panel, (3) 2935 target‐only SNPs from the 3K DArTag panel, (4) 9806 target and OTV SNPs from the 5K DArTag panel, and (5) 4884 target‐only SNPs from the 5K DArTag panel. The GRMs calculated using variants from the 3K and 5K DArTag panel amplicons were positively and significantly correlated to the FanaSNP 50K SNP array GRM; r=0.995 (p<2.2e−16, RMSE=0.039) for the target and OTV 3K panel set, r=0.989 (p<2.2e−16, RMSE=0.059) for the target‐only 3K panel set, r=0.995 (p<2.2e−16, RMSE=0.037) for the target and OTV 5K panel set, and r=0.990 (p<2.2e−16, RMSE=0.055) for the target‐only 5K panel set (Figure 4). Similarly, PC1 of the five GRMs ranged between 85.7% (FanaSNP 50K array) and 89.0% (3K panel target‐only SNPs) and the eigenvectors associated with PC1 were also positively and significantly correlated (Figure S3). The strong linear correlation and minimal differences (RMSE) between the 3K and 5K MIP GRMs compared to the FanaSNP 50K array GRM suggest that these new, medium‐density platforms can be applied for genomic prediction, heritability estimation, and other linear mixed model analyses in quantitative genetics to obtain equivalent results at a vastly reduced cost. We did not observe significant divergence in relationship estimates from the 5K to 3K panel target SNPs, and the inclusion of OTVs produced marginally closer estimates to the 50K array marker relationships for both the 3K and 5K panels.

### 3.6 A modular genotyping platform for genomic selection, genetic identification, and other molecular breeding applications

**FIGURE 5:**
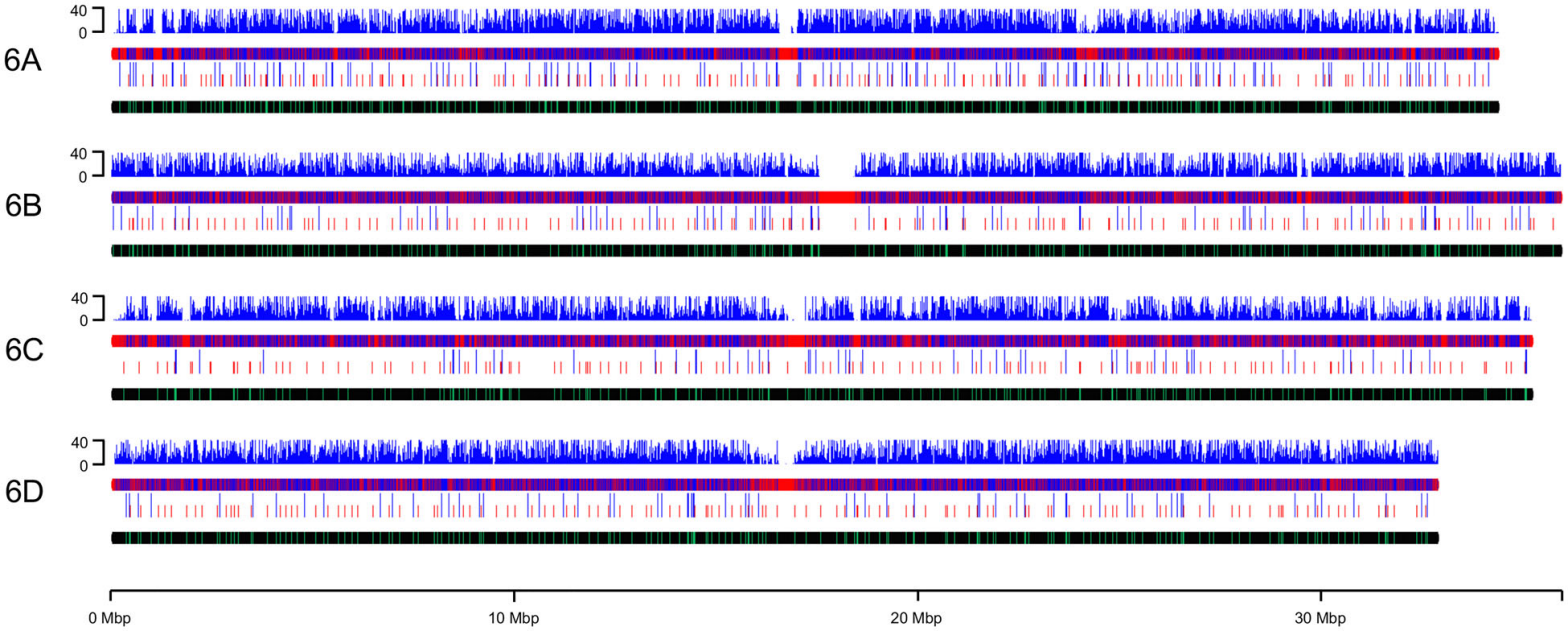
Chromosome‐wide distribution of candidate single nucleotide polymorphisms (SNPs) with corresponding MIPgen probe designs anchored to the “UCD Royal Royce” (FaRR1) cultivate strawberry reference genome having logistic scores above 0.70, visualized as the number of available candidate SNPs with corresponding molecular inversion probes (MIPs, up to 40) per 20 kb window (track 1) and the locations of selected 3K panel DArTag probes (red) and 5K panel exclusive DArTag probes (blue) (track 2). Distributions are shown for subgenomes A, B, C, and D homeologs of ancestral chromosome 6.

The recent availability of high‐quality reference genomes and sequence datasets for strawberry has provided essential resources for trait mapping, functional genomics, and genetic marker development in this allo‐octoploid hybrid species (Bassil et al., 2015; Edger et al., 2019; Hardigan et al., 2020, 2021; Lee et al., 2021; Hardigan et al., 2021; Mao et al., 2023; Shirasawa et al., 2021). The growing molecular breeding toolbox for strawberry has nevertheless needed cost‐effective high‐throughput marker genotyping solutions to enable genomic selection and routine forensic identification of individuals and parents on a large scale (Gezan et al., 2017; Jiménez et al., 2023; Osorio et al., 2021; Pincot et al., 2020, 2021; Whitaker et al., 2020). MIP‐like technologies offer the advantages of high sample throughput and the ability to sequence target amplicon regions at a high read depth, reducing the likelihood of missing genotypes or genotyping errors at heterozygous sites (Wang et al., 2023). The ability to reevaluate the same loci with high read coverage also reduces the need for imputation and supports comparisons of samples across marker datasets. Another benefit of this technology lies in its modularity: Users may add or exchange probe designs to fit their needs, making it possible to produce future iterations of the existing DArTag panels that better represent their own core germplasm diversity, or add specific probes at predictive loci (e.g., R‐genes) to perform MAS and genomic prediction with a single platform (Wang et al., 2023). We were able to identify candidate SNPs supporting designs with high probabilities of subgenome‐specific probe targeting across most of the FaRR1 physical genome landscape, demonstrating the abundance of targets that can be assayed with the technology (Figure 5; Figure S4). The DArTag genotyping platform offers a fast and scalable approach to mid‐density genotyping of hundreds to thousands of individuals with user‐defined DArTag panels.

Avoiding high‐dosage or variable‐dosage marker genotyping is ideal for downstream ease of use for forward and quantitative genetic analyses and user access to diploid analysis pipelines for strawberry research, as well as reducing the likelihood of confounding homeologous alleles that are independently inherited. By evaluating key metrics such as sequence uniqueness and OTV polymorphism in FaRR1 genomic regions flanking validated SNP sites, we were able to identify thousands of candidate loci with corresponding designs that preferentially target and amplify individual subgenomes with low amplification bias between the homologous alleles being targeted. This allowed us to design a marker platform that produces accurate diploid‐behaving genotype calls representing homologous allelic diversity across the four independently recombining A, B, C, and D subgenomes of strawberry (Edger et al., 2019; Hardigan et al., 2020, 2021). We still recommend alignment‐based strategies to support accurate genotype calling with amplicon sequencing data for allo‐polyploids, whether using a complete reference genome sequence or a library of pre‐determined haplotype sequences of known subgenome origin. This will reduce the likelihood that off‐target amplification, when it does occur, will layer non‐homologous information from independent loci over the target locus and contribute to genotyping errors via inaccurate reference‐vs‐alternate allele coverages. In addition to high concordance between alignment‐based genotypes and WGS genomic variant genotypes, we found that the mid‐density 3K and 5K DArTag panels produce highly similar kinship estimates to those produced using the 50K SNP array markers. Accurate kinship estimates should support correspondingly high accuracy for predicting genomic breeding values (Habier et al., 2009; Wang et al., 2023). Beyond genotype calling accuracy, the 3K and 5K DArTag panels target SNPs that reflect significant genetic diversity across a range of domesticated strawberry germplasm and should therefore be useful for genomic prediction and forensic identification in most breeding populations.

## AUTHOR CONTRIBUTIONS

**Michael A. Hardigan**: Conceptualization; data curation; formal analysis; funding acquisition; investigation; methodology; project administration; resources; software; supervision; validation; visualization; writing—original draft; writing—review and editing. **Mitchell J. Feldmann**: Conceptualization; data curation; formal analysis; investigation; methodology; resources; validation; visualization; writing—original draft; writing—review and editing. **Jason Carling**: Formal analysis; investigation; methodology; writing—review and editing. **Anyu Zhu**: Investigation; methodology; writing—review and editing. **Andrzej Kilian**: Conceptualization; funding acquisition; investigation; methodology; project administration; supervision; writing—review and editing. **Randi A. Famula**: Investigation; methodology; project administration; supervision; writing—review and editing. **Glenn S. Cole**: Conceptualization; funding acquisition; project administration; resources; supervision. **Steven J. Knapp**: Conceptualization; data curation; funding acquisition; investigation; methodology; project administration; resources; supervision; writing—original draft; writing—review and editing.

## CONFLICT OF INTEREST STATEMENT

Jason Carling, Anyu Zhu, and Andrzej Kilian are employees at Diversity Arrays Technology (DArT), a company commercializing DNA genotyping technologies.

## Supporting information


**Supplemental Figure** 1. Statistics used to evaluate MIP assay performance for the 7742 reported MIP marker assays and selected 3K and 5K subsets. A) Distribution of median sample amplicon coverage. B) Distribution of low‐coverage sample frequency. C) Distribution of concordance between target SNP genotypes estimated by allele counting versus genomic variant VCF produced by aligning amplicons to the FaRR1 genome assembly.

**Supplemental Figure** 2. Statistics used to evaluate the physical genome distribution of markers selected for the 3K and 5K DArTag panels. A) Distribution of markers per megabase estimated for non‐overlapping 1 megabase genome windows. B) Distribution of physical distances between adjacent marker pairs.

**Supplemental Figure** 3. Distribution principal component 1 estimates using different marker panels for n=279 diverse accessions. A) Distribution of principal component 1 estimates using 44,891 FanaSNP markers and 6,017 target + OTV DArTag 3K panel variants. B) Distribution of principal component 1 estimates using 44,891 FanaSNP markers and 2935 target DArTag 3K panel variants. C) Distribution of principal component 1 estimates using 6,017 target + OTV DArTag 3K panel variants and 2935 target DArTag 3K panel variants. D) Distribution of principal component 1 estimates using 44,891 FanaSNP markers and 9806 target + OTV DArTag 5K panel variants. E) Distribution of principal component 1 estimates using 44,891 FanaSNP markers and 4884 target DArTag 5K panel variants. F) Distribution of principal component 1 estimates using 9806 target + OTV DArTag 5K panel variants and 4884 target DArTag 5K panel variants. In all plots, blue points are UC Davis accessions, and gold points are heirloom and wild accessions.

**Supplemental Figure** 4. Chromosome‐wide distribution of candidate SNPs with corresponding MIPgen probe designs having logistic scores above 0.70, visualized as the number of available candidate SNPs with corresponding probes (up to 40) per 20 kb window (track 1), and the locations of selected 3K panel probes (red) and 5K panel exclusive probes (blue) (track 2). Distributions are shown for the 28 pseudomolecules in the FaRR1 assembly.

**Supplemental Table** 1. Screening panel of 376 octoploid Fragaria accessions used to evaluate assay performance.

**Supplemental Table** 2. Performance and quality metrics for assays selected for 3K and 5K panels.

**Supplemental Table 3**. Parental genetic maps generated using 5K panel genotypes for three full‐sib populations.

**Supplemental File 1**. Target SNPs, alleles, and corresponding FaRR1 design regions submitted for assay design in round‐1 (panel 1) and round‐2 (panel 2).

**Supplemental File 2**. DArTag report generated for assays constructed after round‐1 submission.

**Supplemental File 3**. DArTag report generated for assays constructed after round‐2 submission.
**Supplemental File 4**. Variant site database (VCF format) containing target SNPs in the 5K DArTag panel and WGS‐validated OTVs with coverage from 5K MIP amplicon sequences.
**Supplemental File 5**. Variant site database (VCF format) containing target SNPs in the 3K DArTag panel and WGS‐validated OTVs with coverage from 3K MIP amplicon sequences.

## Data Availability

The genomic DNA sequence datasets for 145 octoploid Fragaria accessions used for identifying candidate variants are housed in the NCBI Short Read Archive under BioProject PRJNA578384. The FaRR1 reference genome for “UCD Royal Royce” and associated gene annotations, transcript and peptide sequences, and supplemental files are available on DRYAD (https://datadryad.org/stash/dataset/doi:10.25338/B8TP7G) and Phytozome (https://data.jgi.doe.gov/refine‐download/phytozome?genome_id=701). All supplemental files for the current study, including DArTag marker submissions and amplicon reports, are available on DRYAD (https://doi.org/10.25338/B8GW62).
