# Supplementary material for: A medium‐density genotyping platform for cultivated strawberry using DArTag technology
Source: Plant Genome. 2023 Nov 8;16(4):e20399. doi: 10.1002/tpg2.20399 (PMC12807182; doi:10.1002/tpg2.20399)
Supplement: Supplementary file 1 [file TPG2-16-e20399-s002.pdf]

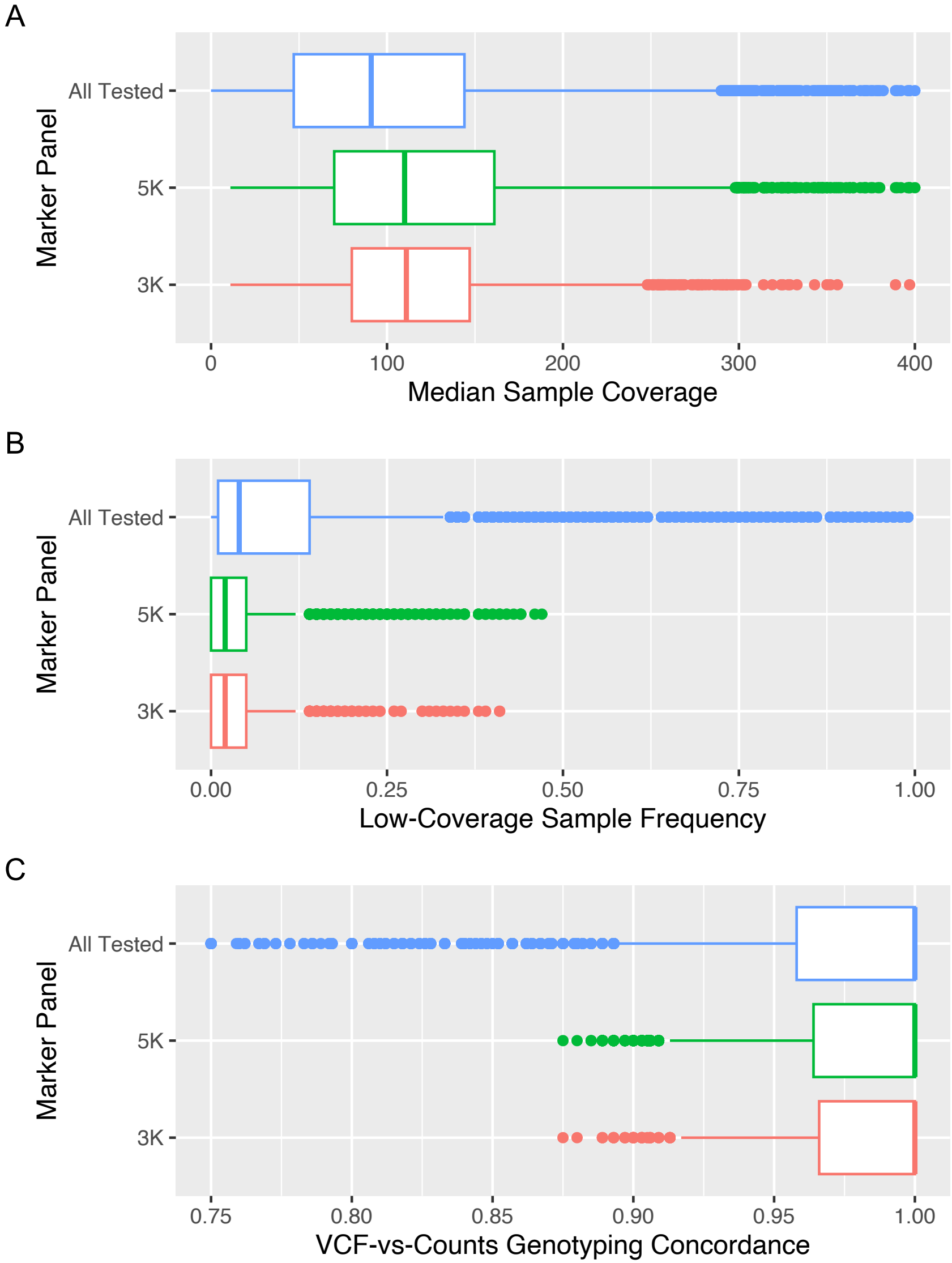
A
Marker Panel
All Tested
5K
3K
0
100
200
300
400
Median Sample Coverage
B
Marker Panel
All Tested
5K
3K
0.00
0.25
0.50
0.75
1.00
Low-Coverage Sample Frequency
C
Marker Panel
All Tested
5K
3K
0.75
0.80
0.85
0.90
0.95
1.00
VCF-vs-Counts Genotyping Concordance
