# Supplementary material for: A medium‐density genotyping platform for cultivated strawberry using DArTag technology
Source: Plant Genome. 2023 Nov 8;16(4):e20399. doi: 10.1002/tpg2.20399 (PMC12807182; doi:10.1002/tpg2.20399)
Supplement: Supplementary file 2 [file TPG2-16-e20399-s005.pdf]

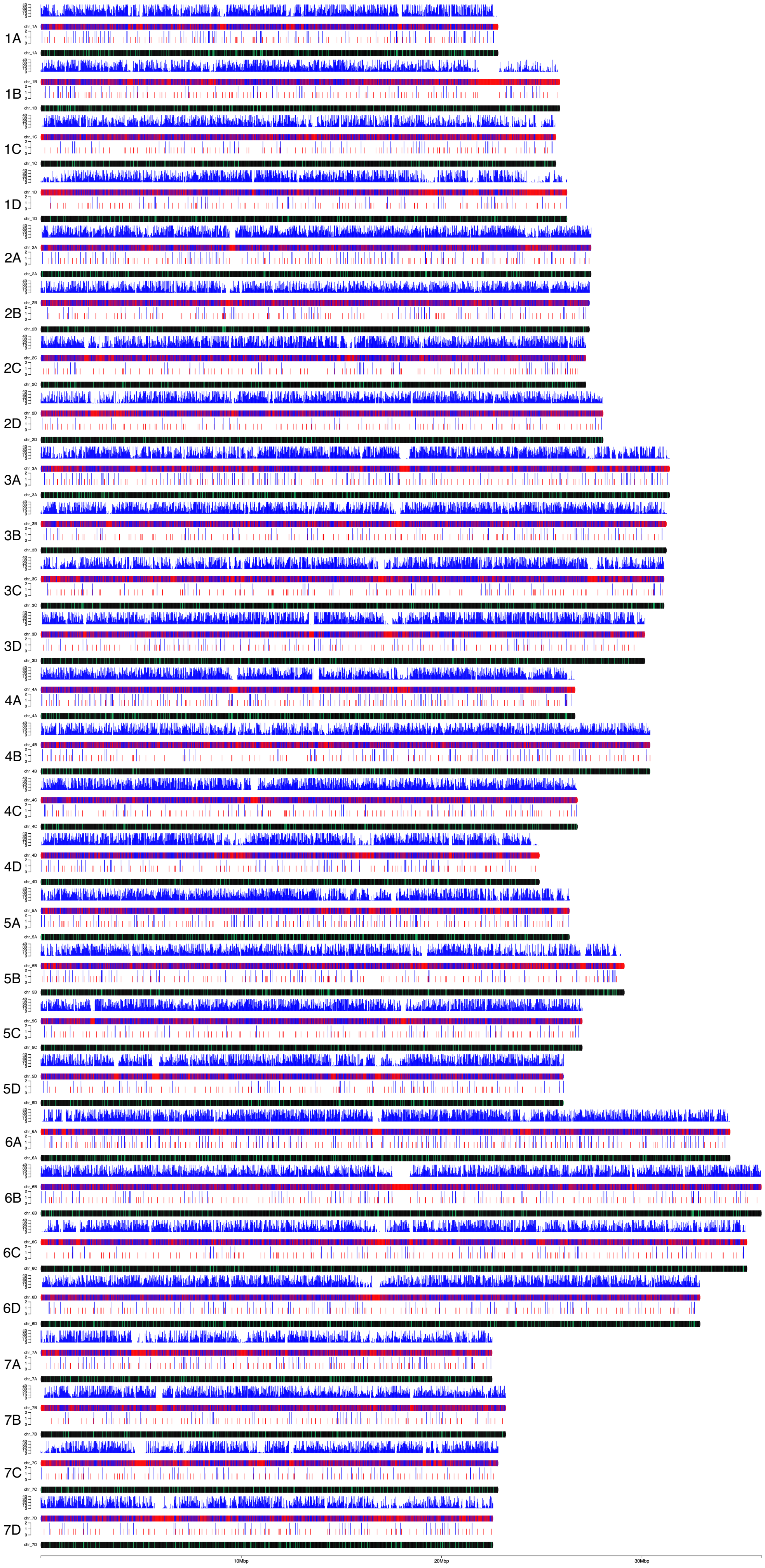
1A
1B
1C
1D
2A
2B
2C
2D
3A
3B
3C
3D
4A
4B
4C
4D
5A
5B
5C
5D
6A
6B
6C
6D
7A
7B
7C
7D
10Mbp
20Mbp
30Mbp
