# Supplementary material for: A medium‐density genotyping platform for cultivated strawberry using DArTag technology
Source: Plant Genome. 2023 Nov 8;16(4):e20399. doi: 10.1002/tpg2.20399 (PMC12807182; doi:10.1002/tpg2.20399)
Supplement: Supplementary file 3 [file TPG2-16-e20399-s006.pdf]

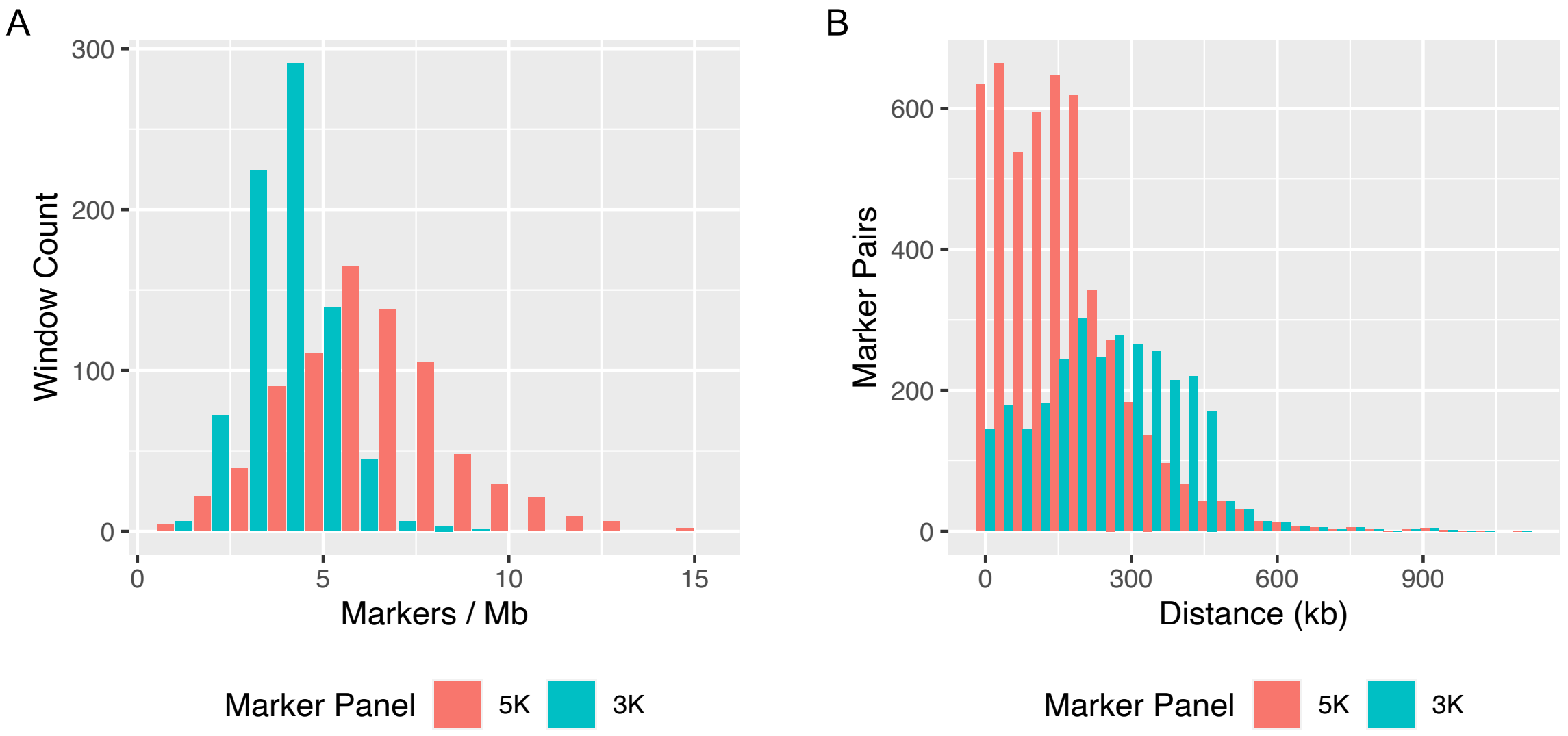
A
Window Count
300
200
100
0
0
5
10
15
Markers / Mb
B
Marker Pairs
600
400
200
0
0
300
600
900
Distance (kb)
Marker Panel
5K
3K
